# Microbiome–Gut–Brain Axis: A Pathway for Improving Brainstem Serotonin Homeostasis and Successful Autoresuscitation in SIDS—A Novel Hypothesis

**DOI:** 10.3389/fped.2016.00136

**Published:** 2017-01-06

**Authors:** Vijayakumar Praveen, Shama Praveen

**Affiliations:** ^1^Kaiser Permanente South Bay, Harbor City, CA, USA; ^2^Providence Little Company of Mary Medical Center, Torrance, CA, USA

**Keywords:** 5-HT, SIDS, gut–brain axis, gut flora, autoresuscitation

## Abstract

Sudden infant death syndrome (SIDS) continues to be a major public health issue. Following its major decline since the “Back to Sleep” campaign, the incidence of SIDS has plateaued, with an annual incidence of about 1,500 SIDS-related deaths in the United States and thousands more throughout the world. The etiology of SIDS, the major cause of postneonatal mortality in the western world, is still poorly understood. Although sleeping in prone position is a major risk factor, SIDS continues to occur even in the supine sleeping position. The triple-risk model of Filiano and Kinney emphasizes the interaction between a susceptible infant during a critical developmental period and stressor/s in the pathogenesis of SIDS. Recent evidence ranges from dysregulated autonomic control to findings of altered neurochemistry, especially the serotonergic system that plays an important role in brainstem cardiorespiratory/thermoregulatory centers. Brainstem serotonin (5-HT) and tryptophan hydroxylase-2 (TPH-2) levels have been shown to be lower in SIDS, supporting the evidence that defects in the medullary serotonergic system play a significant role in SIDS. Pathogenic bacteria and their enterotoxins have been associated with SIDS, although no direct evidence has been established. We present a new hypothesis that the infant’s gut microbiome, and/or its metabolites, by its direct effects on the gut enterochromaffin cells, stimulates the afferent gut vagal endings by releasing serotonin (paracrine effect), optimizing autoresuscitation by modulating brainstem 5-HT levels through the microbiome–gut–brain axis, thus playing a significant role in SIDS during the critical period of gut flora development and vulnerability to SIDS. The shared similarities between various risk factors for SIDS and their relationship with the infant gut microbiome support our hypothesis. Comprehensive gut-microbiome studies are required to test our hypothesis.

## Introduction

Sudden infant death syndrome (SIDS) is defined as a sudden unexplained death in the first year of life in a previously healthy infant, where the cause of death remains unidentified despite thorough investigations including a complete autopsy, death scene investigation, and review of clinical history (1). SIDS is a major cause of postneonatal infant mortality in the western world. In the United States, ~1,500 infants died of SIDS in 2013 alone, despite the steady reduction (1994–2000) in such deaths since the “Back to Sleep” campaign. The incidence of SIDS has remained fairly constant in the last decade, while the rate of other causes of ill-defined, unspecified, and sudden unexpected infant deaths has increased (1, 2). Some infant deaths, which would have been classified as SIDS in the past, are now being classified as resulting from suffocation and asphyxia. The significant reduction in SIDS rate in the past 20 years may be related to increasing diagnoses of other causes of death (1). Japan and the Netherlands have the lowest SIDS rates, at 0.09 and 0.1 per 1,000 live births, respectively, whereas New Zealand has the highest reported SIDS rate (0.8 per 1,000 live births) (3–6). The United States and UK have SIDS rates of 0.57 and 0.41 per 1,000 live births, respectively (7, 8). Prone sleeping position, a significant SIDS risk factor, cannot be easily associated with the other epidemiological risk factors related to SIDS (9).

### Current Hypotheses for SIDS

Sudden infant death syndrome is a condition without a widely accepted singular pathological mechanism.

(1) *Triple-risk model*: this model proposes that SIDS occurs when external stressors simultaneously act upon on a susceptible infant with a vulnerable homeostatic system during a critical developmental period (10).
(2) *Failed autoresuscitation*: animal studies have shown that cardiorespiratory, sleep, and arousal mechanisms are abnormal following exposure to risk factors associated with SIDS or in infants who later succumb to SIDS (11, 12). Although the exact cause of SIDS is unknown, immaturity of brain stem autonomic cardiorespiratory/thermoregulatory control and failure of autoresuscitation during sleep are significant determinants of survival (11, 12). A leading SIDS hypothesis states that a structural/neurochemical brainstem abnormality results in failure of autoresuscitation following exposure to a stressor during a critical developmental period (13, 14). SIDS vulnerability is specific to failed autoresuscitation from an adverse autonomic event (AAE). The initial self-initiated gasp during such an event is dependent of optimal serotonin homeostasis in the brain, which is undermined in SIDS. Imbalance in serotonin homeostasis alters sleep rhythm, thus increasing the chances of AAE (15).
(3) *Medullary serotonergic network deficiency*: SIDS is associated with multiple serotonergic defects including serotonin deficiency (16–19). It has been associated with reduced serotonin in the ventral medulla, pointing to a brainstem-based autonomic dysfunction affecting sleep/arousal/cardiorespiratory reflexes (20–23). Gene polymorphisms related to serotonergic autonomic system may play a role in SIDS (24). In a recent study in neonatal rodents, loss of brain stem 5HT may explain the cardiovascular collapse during apparent severe hypoxic event in some SIDS cases (25). Recent neuropathology studies in SIDS implicate defective neurotransmitter function in the medullary arcuate nucleus, receptor immaturity of the “respiratory center” nucleus tractus solitarius (NTS), and defective function of the serotonergic raphé nuclei of the ponto-medullary ventral median septum and other brainstem serotonergic neurons (26). Abnormalities of the dorsal motor nucleus of the vagus (DMNV) have been associated with SIDS (27). In a significant number of SIDS infants, cerebellar dentate nucleus lesions may represent a developmental susceptibility leading to autonomic cardiorespiratory/arousal dysfunction and sleep-related death when exposed to homeostatic stressors (28). Cummings et al. report that, in addition to respiratory and cardiac dysfunction in normoxemic conditions, neonatal mice with reduced (by 60–70%) brainstem serotonergic neurons from early embryogenesis onward (Pet-1^−/−^) have major defects in autoresuscitation, a life-preserving process utilized by neonatal mammals in severely hypoxic conditions (29–33).
(4) *Neurotransmitters*: neurotransmitter systems (e.g., cholinergic and GABA-ergic) have been shown to be involved in SIDS (34, 35). Reduced muscarinic cholinergic binding in the medullary arcuate nucleus (involved in cardiorespiratory control) has been shown to occur in SIDS (34). GABA neurons in the medulla help regulate homeostasis through interactions with the medullary serotonergic system (35). Significant decrease in GABA A receptor binding was found in the medullary serotonergic system in SIDS cases associated with 5-HT defects (35).

## New Hypothesis

**Figure 1:**
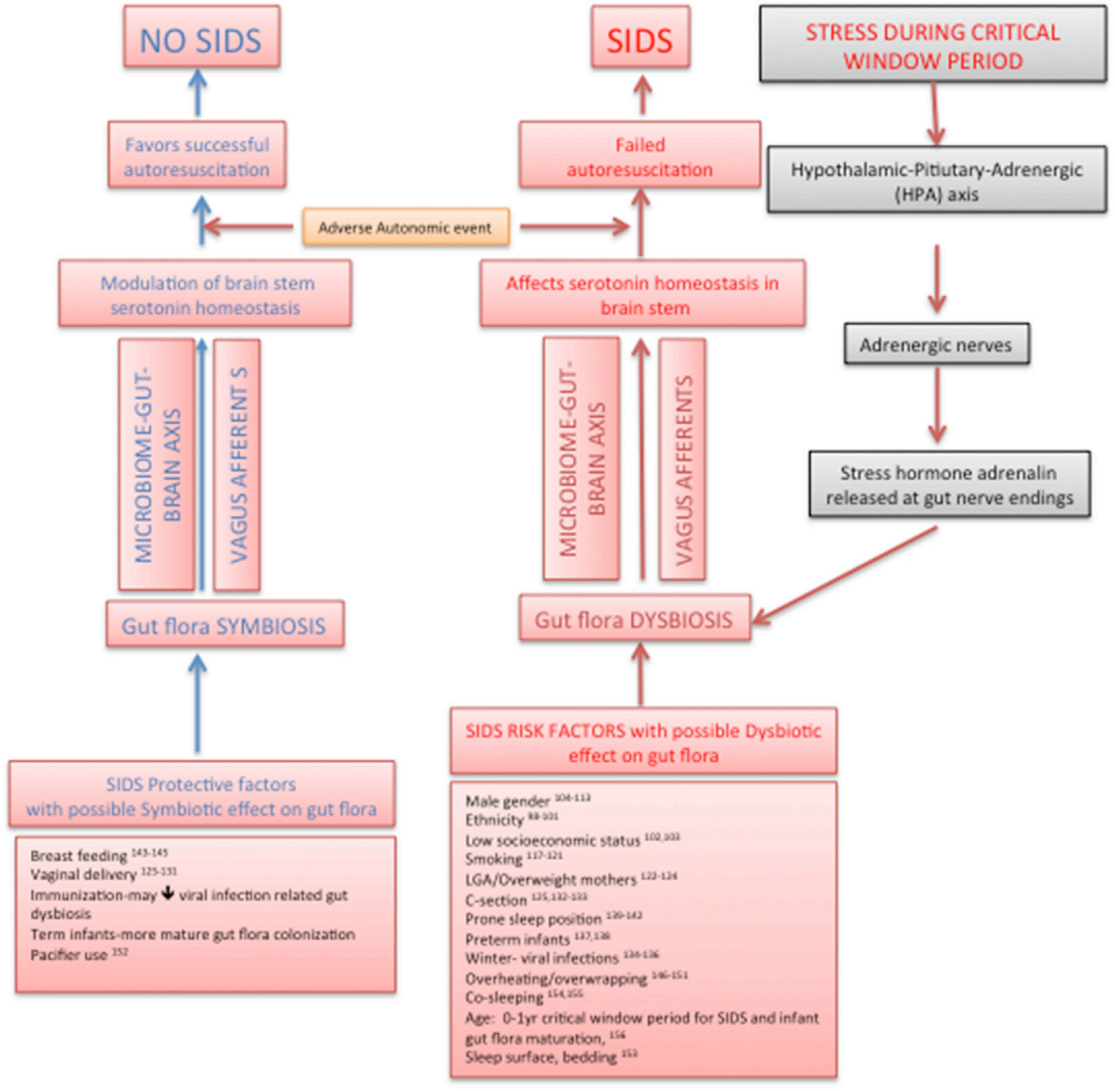
**New sudden infant death syndrome hypothesis pathway: effect on infant gut flora on brainstem serotonin homeostasis and autoresuscitation *via* microbiome–gut–brain axis**.

We propose a new hypothesis that the infant gut microbiome plays an important role in SIDS during the period critical to both gut flora maturation/development and vulnerability to SIDS, by modulating brainstem serotonergic system through the bidirectional microbiome–gut–brain axis, thus tilting the balance in favor of successful autoresuscitation during a sleep-related AAE. The components of our hypothesis, though individually and separately studied in the past, have never been put together as the structure of a SIDS hypothesis. The factors protective against as well as the risk factors of SIDS show some compelling circumstantial evidence of their effects on gut microbiome leading to beneficial and dysbiotic infant gut flora, respectively, with corresponding effects on brainstem serotonergic system. The plausibility of such an SIDS hypothesis would open up a new paradigm for preventative and therapeutic approaches in SIDS. Our hypothesis is the only one till date, which connects the protective/risk factors of SIDS with infant gut flora, their effect *via* microbiome–gut–brain axis on brainstem serotonergic system, and subsequent successful autoresuscitation (Figure 1).

### Microbiome–Gut–Brain Axis

The human adult gut has about 10^14^ microorganisms, 10-fold the human cells and 150-fold the amount of human DNA (36, 37). The human gut microbiome comprises more than 1,000 species, predominantly obligate anaerobes, and includes viruses, protozoa, archaea, and fungi (36–39). Gastrointestinal homeostasis has a significant role in the human general health and well-being (39–41). The concept of the brain–gut axis involves the complex bidirectional homeostatic neuronal communication through the vagus nerve that exists between the central nervous system (CNS) and the enteric nervous system (ENS) (42). Current research studies the mechanism of such communication along this axis and its relationship to normal homeostasis and disease states (42–48). The basic skeleton of the microbiome–gut–brain axis includes gut microbiome, the CNS, neuroendocrine and neuro-immune systems, ENS, sympathetic, and parasympathetic arms of the autonomic nervous system (49). The gut accounts for 95% of the body’s serotonin content. The detailed structure, integration, and functioning of the various components of the above axis have been reviewed extensively elsewhere (45).

Gut microbial colonization also plays a major role in the postnatal development of the endocrine and immune systems, which in turn support CNS function, particularly the developing serotonergic system (41, 42, 50–52). Neurotransmitters, neurohormones, and receptors are ubiquitous in nature, e.g., catecholamines corticotrophin, somatostatin, and GABA derived from bacteria (53–56). Evolutionally speaking, bacteria preceded humans in developing neurotransmitters and recognizing them (57–59). The ontogeny of neurochemicals in mammals has been postulated to arise as a consequence of bacterial lateral gene transfer (60). Thus, the gut microbiota might have played an important role in the evolution of neurodevelopment (61).

#### Gut Vagal Afferents and the Medullary Serotonergic System

Sudden infant death syndrome is associated with multiple serotonergic defects including serotonin deficiency and DMV abnormalities (15–18, 62). We briefly review the vagal afferents, brainstem respiratory neurons, and the medullary serotonergic system.

Vagal afferents outnumber vagal efferents by 10:1, which are sensitive to the paracrine effects of the enterochromaffin cells (ECC), relay through nodose and dorsal root ganglia before synapsing with second-order neurons in the spinal cord, which in turn project into the brainstem. The brainstem has major respiratory neurons concentrated into three recognizable groups comprised of four major nuclei. These include the following: (1) dorsal respiratory group (DRG) centered in the NTS; (2) ventral respiratory group that encompasses the nucleus ambiguous and the nucleus retroambigualis; (3) pre-Botzinger complex (pre-BotC) which contains putative pacemaker neurons; and (4) BotC located in and near the nucleus retrofacialis. The DRG neurons through the phrenic neurons in the cervical spinal cord control the diaphragm.

The medullary serotonergic system projects to brainstem cardiorespiratory nuclei (including the DMNV), cerebellum, and spinal cord, thus modulating cardiorespiratory protective reflexes, central chemoprotective reflexes, arousal/sleep cycles, thermoregulatory reflexes, and maintenance of upper airway patency (63).

Vagal afferents affect respiratory control as shown by altered respiratory pattern after stimulation of visceral vagal afferents in guinea pigs which died within a few hours of bilateral vagotomy; their frequency of breathing significantly decreased within minutes of the procedure (64, 65). Serotonin may regulate developmental brainstem neuronal apoptosis with its pro- or antiapoptotic effects as a result of the receptor sub-family activated (66). Animal studies have shown that the highest density of 5-HT3 receptors are found within the afferent vagal fibers of dorsal vagal complex (67, 68) and vagotomy was found to significantly reduce receptor density (69–71). Stimulation of the NTS 5-HT3 receptors leads to elevation of blood pressure and inhibition of the chemoreceptor-mediated bradycardia and the Bezold–Jarisch reflexes. As an example of sensory neural plasticity, recent rat studies have shown that glucose in the intestinal tract probably induces serotonin release from neuroendocrine cells, which activates 5HT3 on vagal afferent terminals and transmitted centrally (72–79).

### Gut Microbiome Affects the Brainstem

There is emerging evidence from animal and clinical studies on the role of gut microbiome in CNS signaling.

#### Animal Studies

Evidence from rodent studies indicates that the gut microbiome can affect neural development, chemistry, and behaviors, e.g., emotion, pain perception, and stress responses. As rodent gut colonization pattern is similar to humans, they are subjects of choice for gut microbiome studies. CNS tryptophan concentrations are dependent on peripheral content, which suggests that gut flora might play a part in regulating peripheral and central serotonin synthesis (44, 80, 81). TPH2 is responsible for the synthesis of serotonin in brainstem raphe nuclei, which is the origin of most central serotonergic projections (82). Probiotics have been shown to modulate serotonin—a critical central neurotransmitter through multiple strain-specific mechanisms (83). Lyte et al. proposed a “delivery system” by which gut flora can communicate neurochemical messages to the brain. Gut bacteria produce and react to the same neurotransmitters (e.g., serotonin, norepinephrine, dopamine, and GABA) that play a role centrally in modulation of mood (84). Animal studies studying effects of probiotics on CNS function have been extensively and systematically reviewed elsewhere (85). In addition, we have listed few rodent studies looking at the role of pathogenic bacteria and vagus on CNS neurochemistry and behavior (52, 86–92) (Table 1).

**Table 1:** **Animal studies showing effect of gut microbiome/probiotics on the central nervous system (CNS)**.

Reference	Study characteristics
1. Sudo et al. (86)	Participants: mice study, *in vivo*. Germ-free (GF) at 9 weeks of age
	Intervention: stress protocol
	Controls: specific pathogen-free (SPF) BALBc mice, gnotobiotic mice
	Primary outcome: plasma ACTH, corticosterone levels, fecal microflora analysis, plasma cytokine assays
	Conclusion: plasma ACTH and corticosterone responses of GF mice were more susceptible to stress than those of SPF mice. Gut flora regulates the development of the HPA stress response
2. Bravo et al. (87)	Participants: adult male BALB/c mice, *in vivo* (*n* = 36)
	Intervention: *Lactobacillus rhamnosus* 10^9^ cfu gavaged for 28 days
	Control: control broth
	Type of probiotic: *L. rhamnosus* (JB-1)
	Primary outcome: corticosterone level, behavioral analysis, GABA B1b mRNA expression in hippocampus, amygdala, and locus coeruleus
	Conclusion: *L. rhamnosus* supplementation reduced corticosterone response to stress and modulated the GABAergic system in mice. Vagotomized mice did not show the neurochemical effects of this bacterium
3. Desbonnet et al. (88)	Participants: adult Sprauge-Dawley rats (*n* = 20)
	Intervention: *Bifidobacterium infantis* 35624 gavaged for 14 days (*n* = 12)
	Controls: *n* = 8
	Type of probiotic: *B. infantis* 35624
	Primary outcome: corticosterone level, tryptophan and IFN-g, TNF-alpha and IL-6, brain monoamines analysis
	Conclusion: attenuation of pro-inflammatory immune responses and the elevation of the serotonergic precursor, tryptophan, in probiotic-treated group
4. Alenina et al. (89)	Participants: *Tph2*-deficient (*Tph2*^−/−^) mice, *in vivo* study
	Intervention: gene targeting leading to mice with absent TPH2, *n* = 4
	Type of probiotic: none
	Controls: *n* = 6
	Primary outcome: serotonin in the brain of Tph2^−/−^ mice
	Conclusion: the lack of central serotonin in these mice leads to impaired early postnatal growth and altered autonomic control of sleep, thermoregulation, and cardiorespiratory reflexes
5. Lyte et al. (84)	Participants: 9-week-old CF-1 male mice, *in vivo* study
	Intervention: in an animal model of IBD, infection with *Citrobacter rodentium*, to determine whether the infection could lead to anxiety-like behavior
	Controls: saline
	Type of probiotic: none
	Primary outcome: tested for anxiety-like behavior measurement, immune cytokine analysis, and colon for histological analysis
	Conclusion: *C. rodentium* infection could induce anxiety-like symptoms that are likely mediated *via* vagus
6. Gareau et al. (90)	Participants: mouse *in vivo* study
	Intervention: behavior was assessed following infection with the non-invasive enteric pathogen, *C. rodentium* in both C57BL/6 mice and GF Swiss-Webster mice
	Primary outcome: whether daily treatment with probiotics normalized behavior was assessed
	Conclusion: memory dysfunction occurred in infected mice exposed to acute stress, while in the GF setting, memory was altered at baseline
7. McVey Neufeld et al. (91)	Participants: mouse *ex vivo* study
	Intervention: segments of jejunum from 8- to 12-week old GF, SPF, and CONV-GF mice dissected to expose myenteric plexus. Intracellular recordings by impaling cells with sharp microelectrodes
	Type of probiotic: none
	Primary outcome: action potential shapes, firing thresholds, the number of APs fired at 2× threshold, and passive membrane characteristics were measured
	Conclusion: commensal intestinal microbiota are essential for normal excitability of gut sensory neurons. When the vagus nerve is severed, effects of gut bacteria on brain biochemistry, stress response, and behavior disappear
8. Heijtz et al. (92)	Participants: mouse *in vivo* study GF versus SPF mice with normal microbiological gut flora
	Intervention: motor activity and anxiety-like behavior measured
	Conclusion: unstressed GF mice were more active and willing to explore exposed areas of a maze than mice that had normal gut microbiota. Transplanting normal gut bacteria into the GF mice erased those behavioral differences only in early life, suggesting that there is a critical window for gut bacteria to establish normal patterns of behavior
9. Clarke et al. (52)	Participants: male GF animals compared with conventionally colonized control animals
	Intervention: measurement of 5-HT in hippocampus
	Male GF animals have a sex-specific significant elevation in hippocampal 5-HT and 5-HIAA compared with conventionally colonized control animals. Concentrations of tryptophan, the precursor of serotonin, are increased in the plasma of male GF animals, suggesting a humoral route through which the microbiota can influence CNS serotonergic neurotransmission
	Conclusion: microbiome–gut–brain axis in early life modulate hippocampal serotonin levels in a gender-dependent manner

#### Clinical Studies

Emerging evidence from clinical studies in autism indicates a relationship between gut flora and cognitive function. Researchers have reported gut flora dysbiosis with increases in *Clostridium* spp. in autism (93). A probiotic mixture of *Lactobacillus helveticus* and *Bifidobacterium longum* for a month has been reported to decrease anxiety and depression in healthy human (94). Other adult human clinical studies looking at probiotic effects on neurobehavior have been systematically reviewed elsewhere (85).

### Brain Affects Gut Microbiome

Stress induces gut permeability, which allows bacteria/bacterial antigen translocation across the epithelial barrier, thereby activating immune response and resulting in changes in the gut microbiome characteristics (95). Psychological stressors have been reported to modulate infant gut microbiome (47). Prenatal stressors have been reported to cause dysbiosis by decreasing gut Bifidobacteria and Lactobacilli in infant rhesus monkeys (96). In rodent studies, the stress of maternal separation significantly decreased stool lactobacilli on the third day, which returned to baseline by day 7 following separation (97). Stressors acting on an at-risk infant during the critical window period could affect the favorable nature of infant gut flora and consequently affect the brainstem neurotransmitters through bidirectional communication and/or gut barrier function locally.

Based on the evidence from the experimental and clinical studies discussed above, we propose that an optimal (diversity, complexity, and colony counts) gut flora interacts with ECC and modulates (possibly by its serotonin and other paracrine effects) through the afferent vagal endings to the brain stem medullary serotonergic cardiorespiratory centers in infants at risk for SIDS. Recent research in microbiome–gut–brain axis supports role of probiotics to modulate central brain neurochemistry, thus opening up a site for therapeutic targeting for central brain disorders.

#### Shared Risk Factors for SIDS and Gut Dysbiosis

In the following section, we report how each of the protective as well as risk factors for SIDS seems to offer evidence of promoting symbiotic (favorable) and dysbiotic gut (non-favorable) flora, respectively, during the critical window when both SIDS tends to occur and early infant gut colonization is being established.

##### Demographic Factors

(1) *Ethnicity*: studies in indigenous populations have reported a higher SIDS rate compared to the non-indigenous groups within the same countries (98). These differences may reflect differences in maternal smoking, which could affect frequency and density of colonization of infants by potentially pathogenic bacteria and induction/control of inflammatory responses (98). Maternal cigarette smoking and/or alcohol consumption may contribute to abnormal fetal medullary 5-HT development in Native American SIDS infants (99). A recent study has reported diet-related differences in gut flora composition between African-Americans and native Africans. African-Americans had higher levels of 7-α-dehydroxylating bacteria and lower levels of *Lactobacillus plantarum* (which produce methane and is protective against dysbiosis) (100, 101).
(2) *Low socioeconomic status*: SIDS has been associated with lower socioeconomic groups (102). Fecal lactobacilli numbers have been related to socioeconomic status (103). Gut flora differences related to diet, smoking status, and access to health services could be a proxy for lower socioeconomic status.
(3) *Gender*: SIDS shows a male preponderance. Animal studies have shown gender differences in the regulation of serotonergic system (104, 105). Estrogen has been implicated in the modulation of hippocampal serotoninergic system (106, 107). Gender influences gut microbiome (108–111) through unclear mechanisms (110) including hormone–microbe interactions (111, 112) and gender-specific immune responses (113).
(4) *Genetic control*: genes regulating serotonergic network, brain function and development, and cardiac function play an important role in SIDS (114). Studying the role of genetics on gut microbiome is important in understanding the pathogenesis of bacterial diseases (115, 116).

##### Prenatal Risk Factors

(1) *Maternal smoking*: SIDS is five times more common in infants born to mothers who smoked during pregnancy and three times more common in those exposed postnatally to smoking (117, 118). Cigarette smoke exposure and prone sleep position is associated with decreased 5HT1A receptors in the DMNV of SIDS infants (119). A reduction in 5HT1A receptors has been reported in the DMNV of piglets subjected to intermittent hypercapnic hypoxia and nicotine exposure (120). A recent study showed that cessation of smoking improved gut microbial diversity (121). Smoking may play a role in SIDS through its effects on infant gut flora and brainstem serotonin homeostasis.
(2) *Being overweight*: overweight infants and mothers have a higher risk of SIDS (122). Obese human adults had less *Bacteroides* and more *Firmicutes* in their gut flora compared with lean controls (123). A recent review looked at maternal obesity-related pro-inflammatory state and its effect on maternal and *in utero* fetal gut microbiome and development (124).
(3) *Delivery route*: infants delivered by cesarean section have an increased risk of SIDS than those born by vaginal route (125). The mode of delivery has a significant effect on newborn gut flora development (126–128). The gut flora in infants born by cesarean may be altered till 6 months following delivery (129). Prolonged duration of labor during vaginal birth increases the chances of isolation of viable microbes from the stomach and mouth of the infant (130, 131). In addition to exposure to maternal flora, infants born by cesarean section acquire gut flora from their exposure to the immediate environment (132). Aseptic precautions in obstetrics and neonatal units may result in dysbiosis of the infant gut microbiome (133).

##### Postnatal Risks

(1) *Season*: SIDS is more common during winter months (134). There is an association of a viral infection in the days preceding SIDS (135). Stressors such as viral infections during winter may cause dysbiosis in infants (136). Such dysbiosis could play a role in successful autoresuscitation *via* microbiome–gut–brainstem pathway.
(2) *Low birth weight*: the rate of SIDS is higher in low birth weight infants (137). This may be related to the gut colonization patterns of very low birth weight (VLBW) infants compared with normal weight infants. In an elegant study, the initial gut colonization by *Enterobacteria* and *Streptococci* was similar in both VLBW and full-term infants; however, both microorganisms predominated for a longer period of time and the establishment of *Bifidobacterium, Bacteroides, Clostridium*, and *Lactobacillus* was delayed in VLBW infants (138).
(3) *Prone sleep position*: prone sleeping position has been the most important risk factor associated with SIDS (139). In addition to decreased arousal response related to prone sleeping, body temperature seems to be slightly elevated in prone infants (140, 141). Prone sleep position has been associated with *Staphylococcus aureus* gut colonization in SIDS. The increased risk of ingestion/inhalation of bacteria contaminating the sleeping surface during prone position, with resultant gut dysbiosis, could account for the increased risk of SIDS in such infants (22).
(4) *Breastfeeding*: breastfeeding has been shown to be protective against SIDS (142, 143). Breast milk oligosaccharides when fermented by gut flora to form fatty acids results in modulation of infant gut flora. Breast-fed infants show predominant proliferation of *Bifidobacteria* and *Lactobacilli*, whereas formula-fed infants show more *Enterococci* and *Enterobacteria* in their gut flora. In addition, infants who are breast-fed exclusively have been reported to have better sleep arousal patterns than formula-fed infants (144).
(5) *Elevated or reduced room temperature*: overheating of infants has been reported with an elevated risk of SIDS (2, 145). Animal studies have showed that the presence of certain gut flora elevates body temperature of mice and rats. Conn et al. demonstrated that Gram-positive organisms are a major source of the stimulatory effect of gut flora on normal body temperature in mice (146). Body temperature has been shown to have effects on the intestinal flora of hibernating squirrels (147, 148). Oral antibiotics have been shown to reduce nighttime body temperature in rabbits as a result of their effect on their native intestinal flora (149). These studies may help in understanding whether the increased body temperature as a risk factor for SIDS could be a result of aberrant gut flora or vice versa. Elevated body temperature associated with prone sleep position may also play a role in affecting gut flora composition (140, 141).
(6) *Pacifier use*: pacifier sucking has been shown to be strongly associated with the oral colonization of salivary lactobacilli (150). Thus, pacifier use might play a role in favorable oral and subsequently gut flora in infants.
(7) *Sleep surface, bedding, and stuffed toys*: apart from mechanical suffocation and overheating issues, these may act as fomites contributing to the infant gut flora. Sherburn et al. showed that simulated infant head movements and mattress-related factors affect aerial release of bacteria from beds (151).
(8) *Co-sleeping*: recent meta-analyses showed that bed sharing during sleep increases the risk of SIDS, which is further increased when combined with parental smoking, maternal alcohol consumption, and/or drug use (152). The results of a Swedish study suggest that parental skin *S. aureus* establish readily in the infant’s gut, perhaps due to poor competition from other gut bacteria (153). The possible role of acquiring abnormal gut flora from parents/caregivers skin/gut flora by prolonged close contact during bed sharing needs to be investigated further.
(9) *Infant’s age*: SIDS incidence peaks around 2–4 months of the infant’s age, and subsequently decreases by 1 year. Infant gut flora develops through a period of instability in the early months of infancy and reaches more mature adult-like microbiome by 1 year of age, the time by which SIDS disappears. Another condition, not fully explained, affecting the infant during a typical window period is infant colic, in which aberrant gut flora has been recently shown to play a role, amenable to probiotics. Infantile colic is associated with a greater extent with near-miss SIDS infants than among control infants, thereby hypothesizing that colic might play a role as a protective arousal mechanism in such infants (154). From infection/immunity standpoint, this is the time when maternal antibodies reach their nadir making infants more susceptible to infections, including from indigenous pathogenic gut flora. Introduction of supplementary foods around 6 months of age leads to more gut microbial diversity.
(10) *Gestation at birth*: prematurity is associated with a fourfold increased risk of SIDS (137) as well as a dysbiotic intestinal flora, and impaired gut mucosal barrier function and permeability (155–161). *Lactobacillus GG* has been shown to decrease the frequency of *Escherichia coli* K1A translocation in a neonatal rabbit model (162, 163). Extremely preterm newborns (<28 weeks) have a 5- to 10-fold higher incidence of microbial infections than term newborn (164). The preterm neonatal gut colonization is different from that in the healthy, full-term infant gut. Preterm neonates requiring intensive care are colonized by organisms such as Bifidobacteria only gradually and in a delayed fashion. Schwiertz et al. reported similar bacterial colonization patterns in preterm infants in contrast to breast-fed, full-term infants. Bacterial colonization has been observed to be similar in different preterm neonates irrespective of birth weight, feeding regime, and antibiotic therapy. The initial colonization of the newborn GI tract is highly dependent on the environment, and cross-transmission of bacteria is a serious problem in the hospital (165).
(11) *Small for gestational age (SGA)*: it has been hypothesized that SGA infants may have a higher incidence of SIDS as a result of fetal hypoxia-induced decrease in brain serotonergic receptors (16, 166–169).

## Conclusion

We have provided a new SIDS hypothesis whereby the right composition of gut flora in the early critical stage of infant development could possibly optimize or modulate serotonin homeostasis in the serotonergic cardiorespiratory/thermoregulatory brain stem nuclei by a direct communication *via* the vagal afferents as part of the microbiome–gut–brain axis. This may tip the balance in favor of a successful autoresuscitation response to an AAE during sleep. Investigating the role of infant microbiome using newer culture-independent techniques as well as the developmental physiology and neuropathology associated with SIDS may provide more specific strategies than those available currently to define the at risk population. As Hippocrates once stated “All diseases begin in the gut,” research on the gut flora in at risk infants would open new avenues for identifying potential biomarkers and strategies for prevention (e.g., maternal and/or early postnatal probiotic/synbiotic supplementation, diet changes) of SIDS (170).

## Author Contributions

VP was involved in concept and manuscript preparation. SP was involved in editing of the final manuscript.

## Conflict of Interest Statement

The authors declare that the research was conducted in the absence of any commercial or financial relationships that could be construed as a potential conflict of interest.
